# Poncet’s disease in human immunodeficiency virus: a case report

**DOI:** 10.1186/s13104-017-2525-9

**Published:** 2017-06-12

**Authors:** Enoch Omonge, Fredrick Otieno, Mary Kubo, Betty Shiruli

**Affiliations:** University of Nairobi, Nairobi, Kenya

**Keywords:** HIV, Tuberculosis, Reactive polyarthritis

## Abstract

**Background:**

Poncet’s disease is a rare reactive polyarthritis associated with active tuberculosis and no evidence of Mycobacterium tuberculosis in the affected joint.

**Case presentation:**

We report a case of a 35 year old Human Immunodeficiency Virus positive Kenyan male of Kikuyu ethnicity from Kiambu County who presented to hospital with a 6 day history of bilateral knee pain and swelling, bilateral ankle pain with right ankle swelling. The patient reported 6 months history of cough and weight loss. Chest radiograph had features consistent with pulmonary tuberculosis and sputum smear was positive for acid fast bacilli. Analysis of fluid from knee effusion showed no evidence of tuberculosis. Resolution of joint swelling was seen after 3 weeks of tuberculosis chemotherapy suggesting that this was reactive arthritis following pulmonary tuberculosis in a patient infected with human immunodeficiency virus.

**Conclusion:**

This case represents a rare manifestation of tuberculosis presenting as a reactive arthritis. There are very few cases of Poncet’s disease reported in literature and the diagnosis of Poncet’s disease in Human Immunodeficiency Virus/tuberculosis coinfected patient is extremely uncommon. This case report has been presented to raise awareness of this unusual tuberculosis complication and review its diagnosis and treatment.

## Background

Poncet’s disease also known as tuberculous rheumatism is a rare syndrome which was first described in 1897 by the Frenchman Poncet [1]. The disease presents with reactive arthritis due to tuberculosis infection elsewhere from the joint with no evidence of joint bacillary invasion [2, 3].

Rapid and complete remission of arthritis with no permanent articular sequelae occur following tuberculosis chemotherapy. This contrasts with tuberculous septic arthritis where joint destruction is a common association particularly if treatment is delayed [4, 5]. There are very few cases of tuberculous rheumatism reported in literature in patients who are HIV infected [6].

We present a case of reactive arthritis accompanying pulmonary tuberculosis and review literature to help document the salient clinical findings in Poncet’s disease and also distinguish the syndrome from direct tuberculous septic arthritis.

Resolution of this reactive arthritis following tuberculosis chemotherapy takes a few days to weeks as opposed to tuberculosis septic arthritis that has a more protracted course. Morbidity resulting from reactive arthritis would be mitigated with early diagnosis and treatment.

## Case presentation

A 35 year old male of Kikuyu ethnicity from Kiambu, presents with 6 months history of productive cough and progressive weight loss. NN is a 10 pack-year smoker and has history of heavy alcohol consumption for a long time.

At the time of presentation the patient had noticed pain and increasing swelling involving both knee joints and right ankle joint over 6 days. The left ankle joint and both wrists were also painful. The patient reported no diarrhoea, skin rash, genito-urinary symptoms or eye problems. There was no family history of similar illness.

Examination of the patient revealed the following; Temperature 38 °C, respiratory rate 40/min, pulse rate of 104/min. Patient was normotensive, acyanotic and wasted. Examination of the knee joints demonstrated bilateral knee effusion with limitation of movement of both joints. Auscultation of the chest revealed bilateral crepitations.

**Fig. 1:**
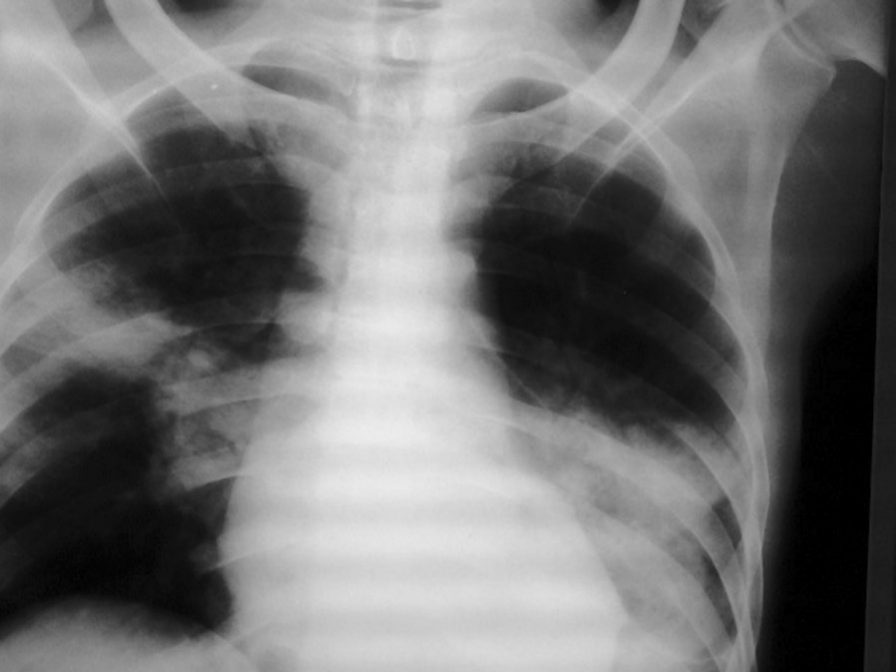
Chest X-ray of NN showing bilateral opacities

Bilateral opacities (Fig. 1) were seen on a postero-anterior chest radiograph. Full blood count revealed leukocytosis, with an Erythrocyte Sedimentation Rate (ESR) of 70 mm/H. HIV test was positive with CD_4_+ count of 109/ml and HIV viral load of 237,000 copies/ml. The patient had no prior knowledge of his HIV status and was naïve to antiretroviral drug therapy. His sputum smear for acid fast bacilli was positive.

His serum Albumin was 24 g/l and INR 1.57. Other liver function tests were normal. The patient had features of acute kidney injury which abated on rehydration.

His antibody test for salmonella and brucella were both non-reactive. Hepatitis B and C markers were negative. Venereal Disease Research Laboratory (VDRL) was non-reactive for syphilis, while serum uric acid was normal. Anti-CCP (cyclic citrullinated peptide), antinuclear antibody and rheumatoid factor were all negative. Urine examination was normal.

Synovial fluid was reported as cloudy fluid with 17,000 cells: 74% neutrophils, 10% lymphocytes and 12% monocytes. No birefringent crystals were seen in the synovial fluid which was also sterile on culture and negative for tuberculosis.

The patient was treated with rifampicin 10 mg/kg/day, isoniazid 5 mg/kg/day, ethambutol 15 mg/kg/day and pyrazinamide 20 mg/kg/day and within 2 weeks reported resolution of pain and joint swelling.

After 2 months of therapy, the patient had complete remission of joint symptoms and had gained weight. He was commenced on highly active antiretroviral therapy (HAART) following the initial intensive phase of tuberculosis chemotherapy.

## Discussion

Resurgence of tuberculosis worldwide has been seen in the era of HIV infection. High prevalence of tuberculosis is seen in developing countries with extrapulmonary manifestations comprising a significant proportion. Bone and joint disease constitute 1–3% of all cases of tuberculosis [7] and is the fourth most common cause of extra-pulmonary tuberculosis after pleural, lymphatic and genitourinary system involvement [8]. Direct musculoskeletal Mycobacterium tuberculosis infection manifests as spondylosis, osteomyelitis, septic arthritis or tenosynovitis.

Tuberculous septic arthritis tends to be monoarticular with X-ray images showing enlargement of articular space, effusion and pannus. Cartilage destruction and subchondral bone erosion is also seen. Tuberculous septic arthritis has slow response to treatment and remission may be incomplete with residual joint damage [7]. Reactive arthritis has not been a common feature of HIV associated musculoskeletal disorder.

Like in HIV infection, the use of corticosteroids, immunosuppressant and biologics can trigger reactivation or dissemination of tuberculosis [7, 9]. Anti tuberculosis drugs may induce tendinopathies and lupus like syndrome [10].

**Fig. 2:**
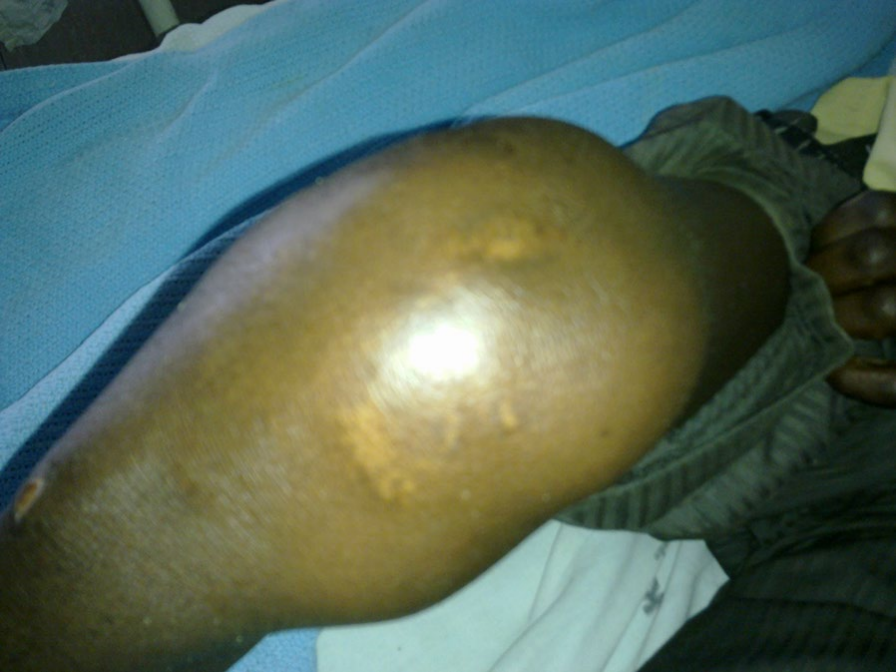
Knee effusion in patient NN with sterile polyarthritis and pulmonary tuberculosis

Literature records aseptic polyarthritis (Fig. 2) in presence of active tuberculosis as a rare immunologic manifestation of tuberculosis with rheumatic presentation. This occurs mainly in patients with extrapulmonary tuberculosis and the consequent reactive arthritis may be associated with other immunologic manifestations such as Erythema nodosum [1, 7]. About 50% of these patients have associated pulmonary tuberculosis [11].

This non-tuberculous reactive arthritis developing following a distant infection as seen following a primary pulmonary portal in our patient, has been described to preferentially affect young adults [12]. Large weight bearing joints disease is the typical presentation as was the case of bilateral knee and ankle joint involvement in our patient [11].

The mechanism of Poncet’s disease is still incompletely understood. Molecular mimicry and heat shock protein have been hypothesized to explain the aetiopathogenesis of Poncet’s disease [10, 13]. It is thought that Mycobacterium tuberculosis induces a T cell mediated cross reactivity between its antigens and cartilage proteoglycan. Alternatively patients with or without HIV may exhibit hypersensitive immune response to mycobacterial antigen. Another possible explanation is reactive arthritis as an immunological sequelae of HIV-induced decreased peripheral T cell response against arthritis-associated bacteria and resultant cytokine imbalance.

Genes located within the MHC may play a role in susceptibility to Poncet’s disease. A Mexican study showed increased association with human leukocyte antigen (HLA)-B57 and DQBI*0301. Further, patients with HLA-DR3 and HLA-DR4 manifest brisk T-lymphocyte mediated response to mycobacterial antigen and also show greater predisposition to tuberculosis rheumatism [14–16]. In Caucasians, unlike African patients, a strong association between Poncet’s disease and HLA B27 has been found. HLA B 27 studies were however not done in our patient. Studies in African patients document absence of HLA B27, and possible HLA B14 association to reactive arthritis [17].

Novaes et al. propose criteria to improve the diagnosis of Poncet’s disease [18]. This criterion includes evidence of active extra-articular tuberculosis, rheumatic manifestation of more than one joint, absence of personal and family antecedent, as well as lack of axial vertebral column and sacroiliac impairment. In addition, unspecific laboratory findings, complete remission of the rheumatic manifestation with antituberculous chemotherapy, no permanent articular sequelae, and exclusion of other rheumatic diseases are significant.

Modern diagnostic methods including Polymerase Chain Reaction (PCR) gene-Xpert may increase accuracy and ease of diagnosis. Our patient adequately met the criteria of diagnosis of Poncet’s disease. A synovial biopsy would enhance the exclusion of tuberculous septic arthritis. However synovial biopsy may lead to complications such as fistula formation.

Reactive arthritis occurring after infection elsewhere in the body would have been a plausible differential diagnosis in this patient. Commonly associated infections include Yersinia, salmonella, shigella and campylobacter [19]. Our patient had no genitourinary or gastrointestinal tract infections antecedent to the arthropathy. Enthesitis, mucocutaneous features, and circinate balanitis seen in reactive arthritis was not also seen. Diarrhoea, urethritis and conjunctivitis typically manifest in Reiter’s syndrome was not observed, suggesting likely association with established pulmonary tuberculosis.

An alternate differential diagnosis is HIV associated arthropathy. This presents as an asymmetric oligoarthritis predominantly in males, with involvement of knee and ankle joint or as a symmetric polyarthritis resembling rheumatoid arthritis. This has been postulated to be due to direct arthritogenic effect of the HIV infection [20]. The diagnosis of pulmonary tuberculosis and subsequent response to anti-tuberculosis therapy makes HIV-associated arthropathy unlikely in this patient.

In hind sight we note that this patient responded to treatment with anti-tuberculosis drugs with rapid remission and complete resolution in 2 months. The presence of established smear positive tuberculosis in a young male with no antecedent history of a rheumatic disorder, and polyarticular presentation with laboratory evidence neither suggesting tuberculosis septic arthritis nor other rheumatic conditions makes the diagnosis of Poncet’s disease almost certain. Synovial tissue biopsy in this case would not have altered the therapeutic discourse.

## Conclusion

This case represents a rare manifestation of tuberculosis presenting as a reactive arthritis. In a high burden tuberculosis and HIV infection setting, early recognition would help forestall complications resulting from delayed treatment. There are very few cases of Poncet’s disease reported in literature and the diagnosis of Poncet’s disease in HIV/tuberculosis coinfected patient is extremely uncommon. This case report has been presented to raise awareness of this unusual tuberculosis complication and review its diagnosis and treatment.

## Abbreviations

CCP: cyclic citrullinated peptide
ESR: Erythrocyte Sedimentation Rate
HAART: highly active antiretroviral therapy
HIV: human immunodeficiency virus
HLA: human leukocyte antigen
PCR: Polymerase Chain Reaction
VDRL: Venereal Disease Research Laboratory
